# Supplementary material for: Latent periodic process inference from single-cell RNA-seq data
Source: Nat Commun. 2020 Mar 18;11:1441. doi: 10.1038/s41467-020-15295-9 (PMC7080821; doi:10.1038/s41467-020-15295-9)
Supplement: Supplementary file 1 — Supplementary Information [file 41467_2020_15295_MOESM1_ESM.pdf]

Latent periodic process inference from single-cell RNA-seq data

Liang et al.

Supplementary Figures

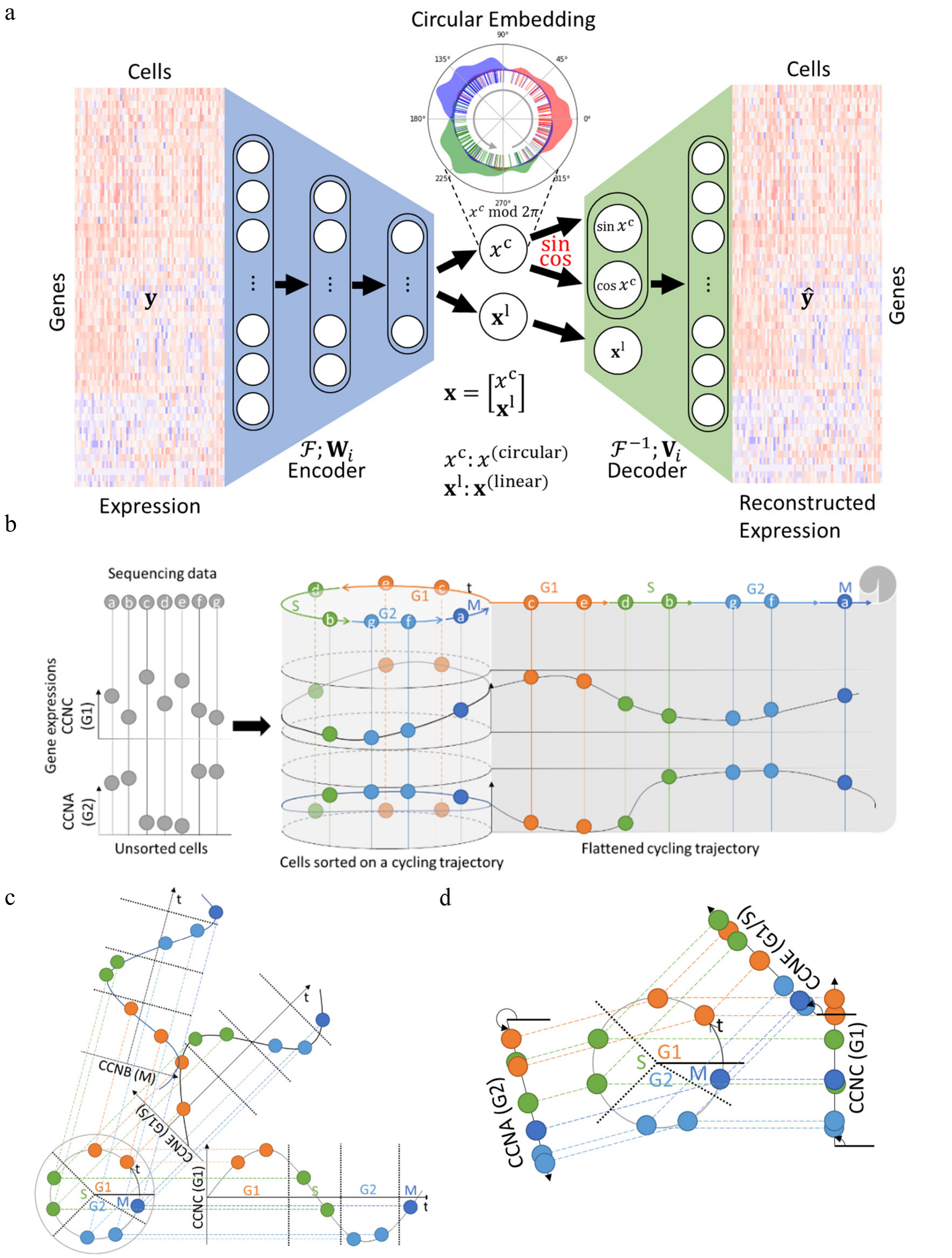

Supplementary Figure 1 related to Fig. 1: Mechanism of Cyclum. **a)** The diagram of the auto-encoder. Variables are defined in Methods, with subscript n omitted for simplicity. **b)** The unraveling of the periodic pseudo-time. In the sequencing data, cells are unsynchronized. Cyclum infers a real-value timing "coordinate" for each cell on a circle. The expressions of genes are assumed to be periodic and follow a roughly sinusoidal pattern through the time. The gene expression can be viewed as the projection of the cycle onto an axis. The angle of the axis is the phase of the gene (i.e. peak timing in radian measure). **c)** The optimal sinusoidal representation of Cyclum. **d)** The optimal sinusoidal representation of Cyclum, without showing timings for genes.

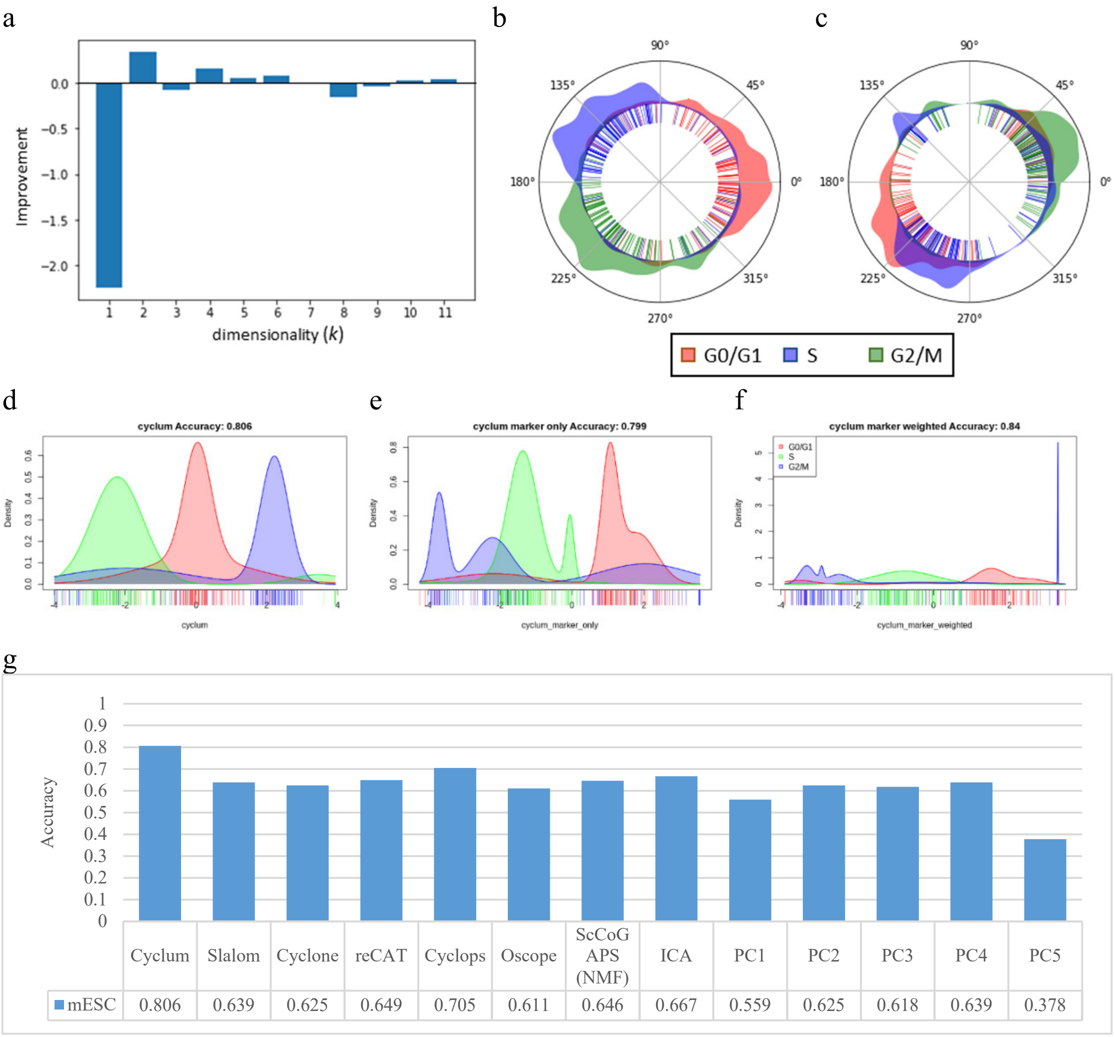

Supplementary Figure 2 related to Fig. 2: Additional results on the mESC dataset.
a) Improvement in fitting (i.e., relative difference in MSE) of Cyclum, compared with PCA, using the same dimensionality (k). X-axis shows the number (k) of dimensions and Y-axis shows the relative improvement in fitting, where higher is better.
b, c) Circular embedding of Cyclum with dimensionality $k = 2$ (b) and 1 (c), the circular dimension included. Colors correspond to cell-cycle phases. Curves are empirical distributions for the stages and the lines represent individual cells. Accuracy evaluated against flow-sorting is 0.806 and 0.639, respectively.
d, e, f) Utilizing marker genes as prior knowledge in Cylcum. The panels are for (d) no prior knowledge, (e) only keep marker genes, and (f) set more weights on the marker genes. Curves are empirical distributions for each phase and the vertical lines beneath represent individual cells. Accuracies are 0.806, 0.799 and 0.840, respectively.
g) Cell-cycle classification accuracies of thirteen methods on the mESC dataset.

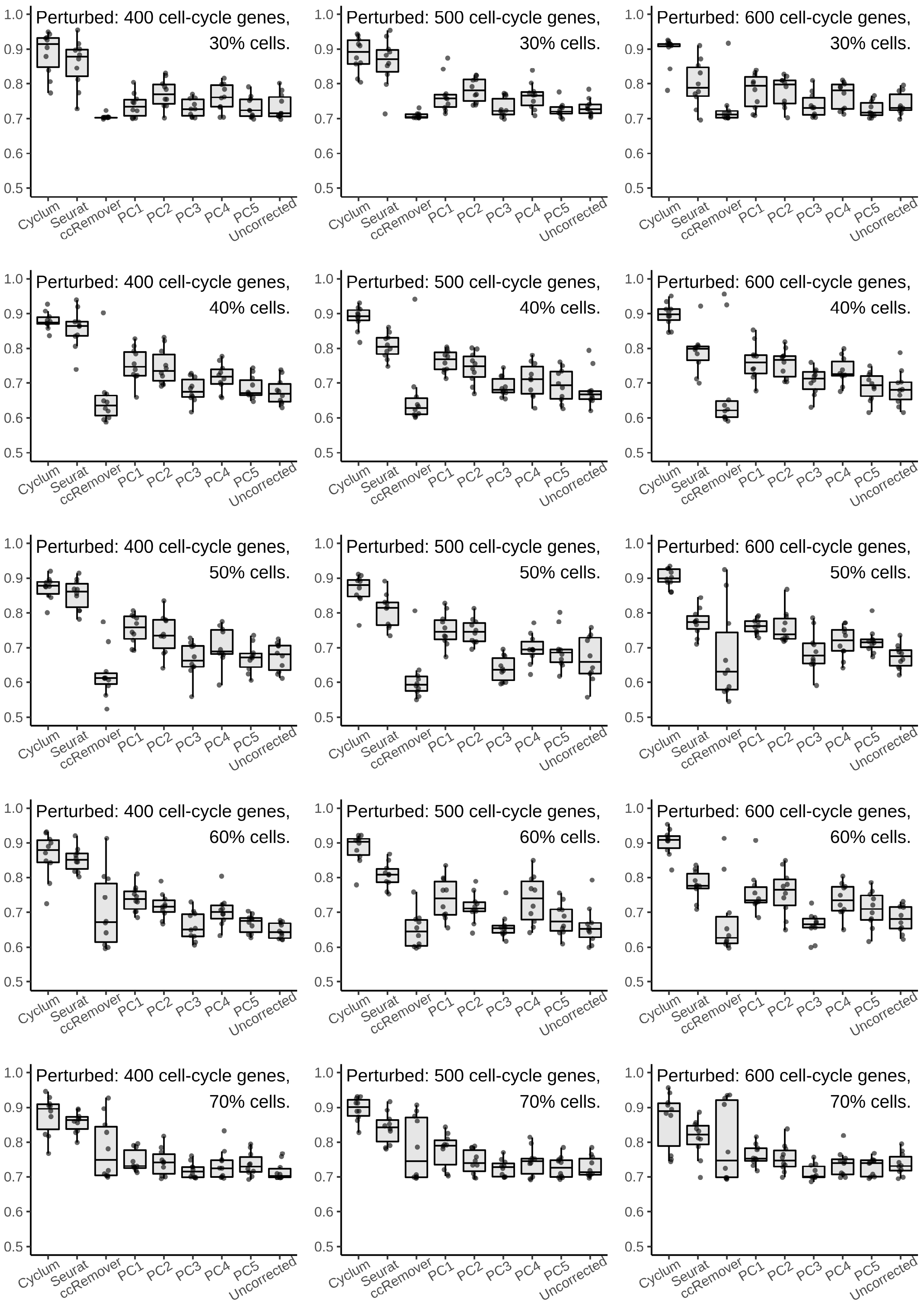

Supplementary Figure 3 related to Fig. 3: Each boxplot show median (center lines), interquartile range (hinges), and 1.5 interquartile range (whiskers) of $n = 10$ randomly generated virtual tumor datasets using data corrected by Cyclum, Seurat, ccRemover, Principal Component (PC) 1-5, and the uncorrected data. The columns are for perturbing 400, 500, and 600 cell-cycle genes and the rows are for perturbing 30%, 40%, 50%, 60%, and 70% of the cells to form a new subclone. The number of other genes perturbed is fixed to 1000. Cyclum performs significantly better when more cell-cycle genes are perturbed (second and third columns). Model-based construction leads to this improvement.

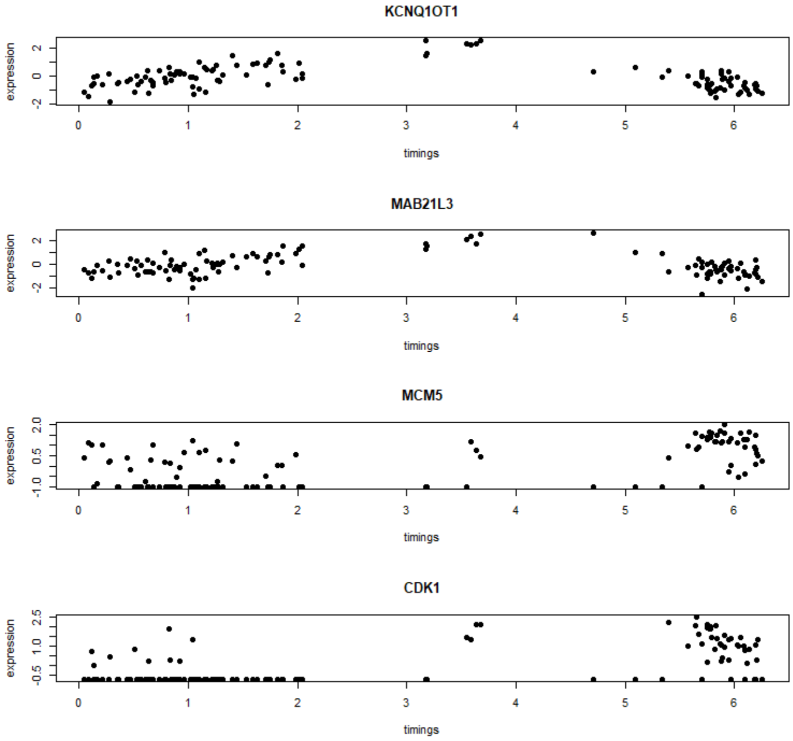

Supplementary Figure 4 related to Fig. 4: Top genes found by Cyclum compared with known cell cycle genes. These plots show the correlation of the expressions (Y-axis) of the top genes (*KCNQ1OT1*, *MAB21L3*, *MCM5*, *CDK1*) found with inferred pseudo-time (X-axis). *MCM5* and *CDK1* are common cell-cycle marker genes. The non-coding RNA *KCNQ1OT1* is related to (malignant) cell proliferation[1–4] and *FBLIM1* is a marker of malignancy[5,6].

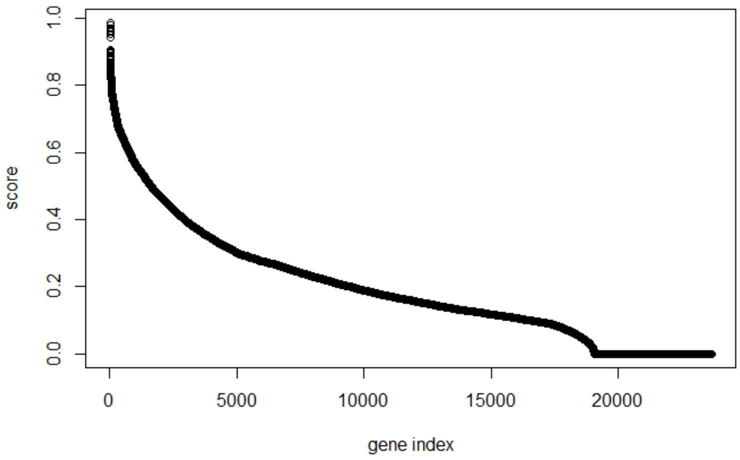

Supplementary Figure 5 related to Fig. 4: Cyclum assigned cycling scores for the genes. A gene with the rank of x, shown on the X-axis, has the score y, shown on the Y-axis. Genes with dramatically higher scores are potential marker genes.

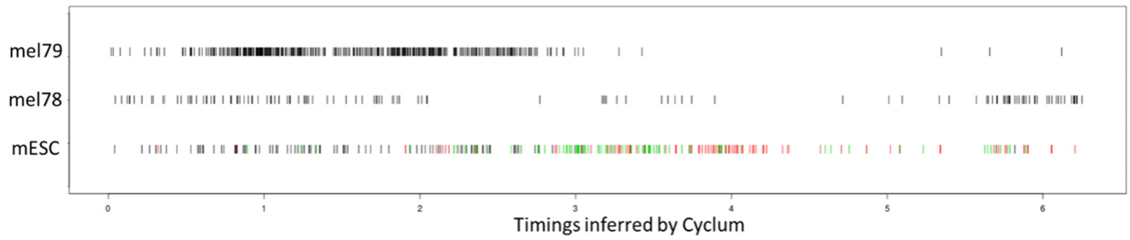

Supplementary Figure 6 related to Fig. 4: Comparison of pseudo-times. This chart shows the comparison of pseudo-times (X-axis) inferred on mESC, mel78, and mel79 (Y-axis). Stages of mESC cells (active cycling) are encoded in color (Black: G0/G1; green: G2/M; red: S). Stages of mel78 (partially cycling) cells and mel79 (almost non-cycling) cells are unknown. For actively cycling mESC cells, the cells are relatively uniformly distributed over the pseudo-time axis. Mel78 contains about 40% cycling cells, most cells cluster in time range $(0, 2)$, which may be G0/G1. G2/M may be in $(3, 4)$ and S may be in $(5, 2\pi)$. For Mel79, which reportedly is not under active cycling, almost all cells are clustered between $(0, 3)$ while the interval $(3, 2\pi)$ is almost vacated.

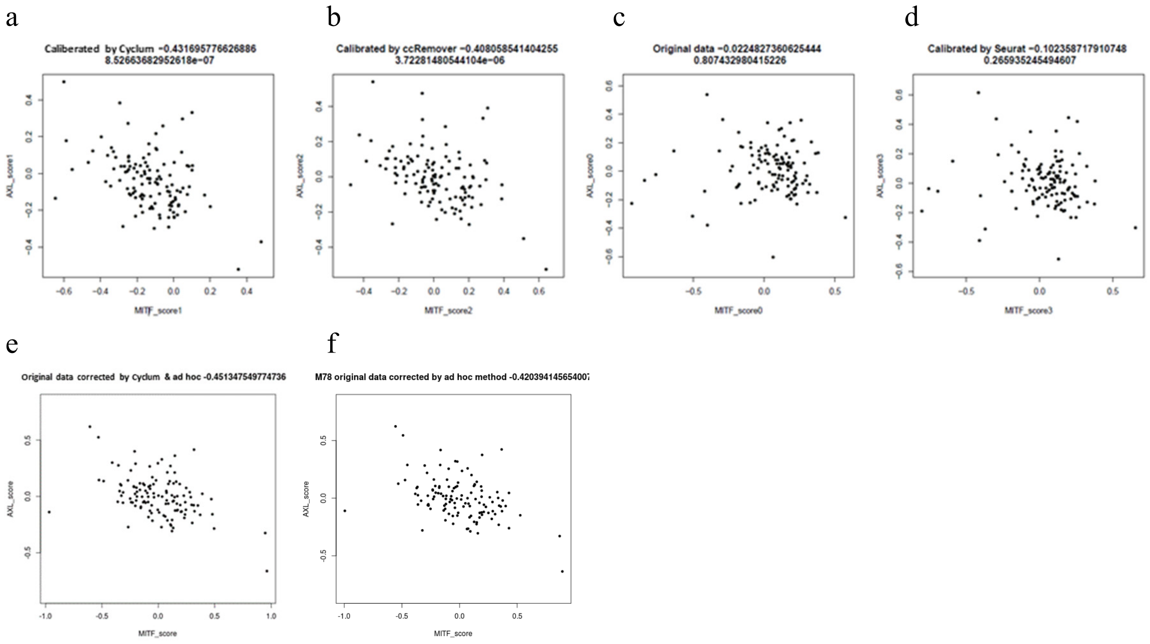

Supplementary Figure 7 related to Fig. 4: AXL and MITF program. For a-d, the correlation of MITF score (X-axis) and AXL score (Y-axis) on expressions corrected by **a)** Cyclum, **b)** ccRemover, and **d)** Seurat, compared with **c)** the uncorrected original expressions. For (e) and (f), the expressions corrected by **e)** both Cyclum and the ad hoc method. **f)** only the ad hoc method suggested in the original report.

a

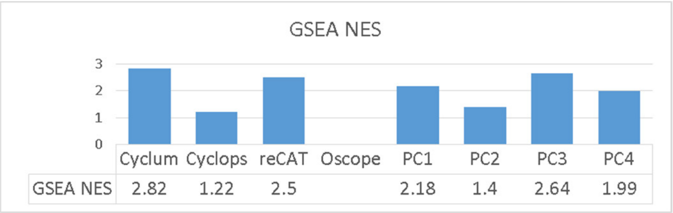

b

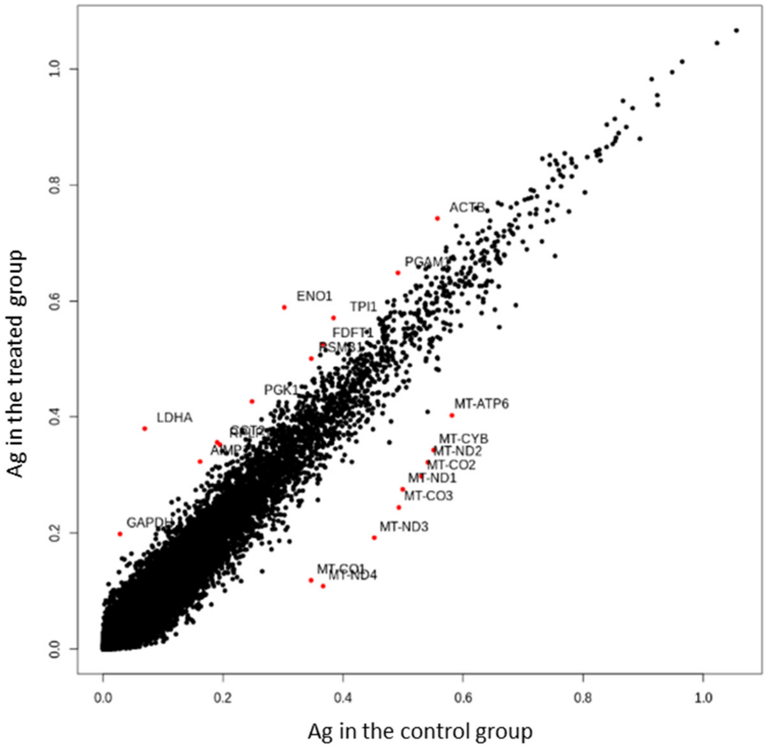

Supplementary Figure 8: Results on hESC 10X dataset.
a) Comparison of GSEA normalized enrichment score (NES) of the methods on hESC 10X dataset. Score for Oscope is not available as it fails to return a result within 100 hours.
b) Comparison of the magnitudes (Ag) between the nicotine treated group and the control group. Each dot represents a gene, whose x-coordinate is its circular magnitude in the control group, and y-coordinate is that in the nicotine treated group.

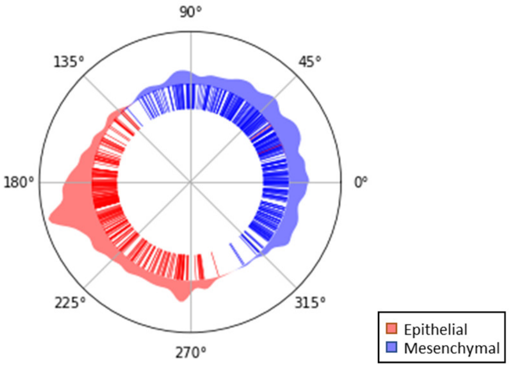

Supplementary Figure 9: Result on EMT dataset. Embedding of MET-EMT circular pseudotime in a preliminary experiment. Colors corresponds to two cell types labelled in the original study via the expression levels of the known EMT/MET genes. Curves are empirical distributions for each phase and the lines represent individual cells.

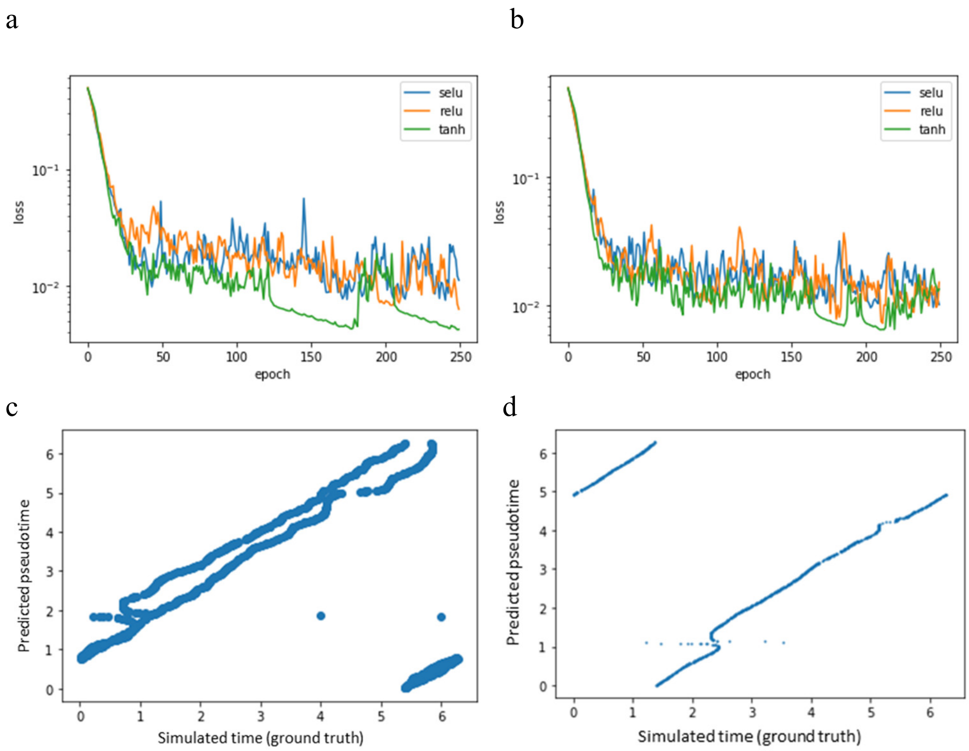

Supplementary Figure 10: Simulation results supporting the robustness of Cyclum to additional non-linearity and choice of using tanh as the activation function.

a, b) Comparison of activation functions on two simulated sinusoidal datasets. X-axis: number of training epochs. Y-axis: mean squared error loss of fitting.

c) Using linear and circular components to decipher combination of sigmoid and sinusoid. X-axis: simulated time, the ground truth; Y-axis: inferred pseudotime; both mod 2π. A diagonal pattern indicates good match of the predicted and the ground truth. A circular translation on the predicted pseudotime is considered normal (i.e., the portion under Y = 0.8 may be cut and paste to the top of the figure to form a complete diagonal).

d) Using circular components to decipher sinusoid. X-axis: simulated time, the ground truth; Y-axis: inferred pseudotime, both mod 2π. A diagonal pattern indicates good match and translation is normal.

Supplementary Tables

Supplementary Table 1: Enrichment Area-under-Curve on Melanoma

Patient	Cyclum	PC 1	PC 2
#79	0.641	0.594	0.529
#81	0.602	0.616	0.565
#80	0.649	0.639	0.569
#78	0.649	0.626	0.623
#88	0.585	0.543	0.527

We count the numbers of known cell-cycle genes in the top g proposed genes. We vary g from one to the total number of genes (i.e. $g = 1 \dots G$). The resulting number of cell-cycle genes changes accordingly. Enrichment Area-under-curve (AUC) is defined as the sum of all the resulting numbers of cell-cycle genes over the g's.

Supplementary Table 2: The proposed top genes

Gene	Weight	Gene	Weight	Gene	Weight	Gene	Weight
KCNQ1OT1	0.986	*GSTO1*	0.849	*COPS8*	0.813	*EIF5A*	0.778
MAB21L3	0.979	*MTRNR2L4*	0.844	*LSM10*	0.812	*TRMT112*	0.778
LOC100131257	0.970	*TSTD1*	0.843	*ARF4*	0.810	*SOD1*	0.777
UGDH-AS1	0.960	*S100A11*	0.842	*BLVRB*	0.809	*MIF*	0.777
FBLIM1	0.960	*ATCAY*	0.842	*PGAM1*	0.807	*MS4A10*	0.775
ORC4	0.954	*NLRP12*	0.836	*PSMA2*	0.806	*KPNA2*	0.775
ABCC9	0.941	*ATP5G1*	0.836	*PKMYT1*	0.804	*HADHB*	0.774
TLCD2	0.904	*TSIX*	0.835	*NDUFA8*	0.804	*ROMO1*	0.774
SF3B14	0.902	*RPL3*	0.835	*TPTE2P1*	0.800	*ALG8*	0.773
REXO1L1	0.899	*ARHGEF26-AS1*	0.832	*ASTN2*	0.800	*MLANA*	0.773
C21orf62	0.893	*CHMP2A*	0.831	*COX7A2L*	0.800	*ZBTB8A*	0.772
HYDIN2	0.889	*COA4*	0.830	*S100B*	0.798	*SDCBP*	0.772
ATP5G3	0.885	*COX6C*	0.829	*NDUFB10*	0.795	*TUBB*	0.772
LOC646214	0.879	*RPL29*	0.826	*PSMD8*	0.793	*LGALS3*	0.770
FTH1	0.878	*GSTP1*	0.826	*LINC00346*	0.790	*CCBE1*	0.770
ANKRD20A9P	0.878	*IKZF3*	0.824	*ADIPOR1*	0.786	*HNRNPM*	0.769
RPS3	0.878	*SPN*	0.824	*ATOX1*	0.785	*MTRNR2L6*	0.768
PRKAR1A	0.876	*PDDC1*	0.823	*SLC25A3*	0.785	*ANXA5*	0.768
SLC25A6	0.866	*DBI*	0.820	*KLRD1*	0.783	*PARK7*	0.768
SHISA9	0.866	*ODF2L*	0.819	*MTRNR2L10*	0.783	*UHRF1*	0.768
PSMB4	0.861	*ATP5A1*	0.819	*SRP72*	0.782	*CD84*	0.767
FABP5	0.859	*COX7A2*	0.819	*TXN*	0.781	*SNRPE*	0.767
NME1	0.857	*C1QBP*	0.818	*PYCARD*	0.780	*PAICS*	0.767
LOC643406	0.855	*ARGFX*	0.815	*NDUFS7*	0.779	*COX6B1*	0.767
LAMTOR1	0.850	*OST4*	0.815	*NDUFB3*	0.778	*RPL8*	0.767

Supplementary Table 3: The melanoma datasets

Patient	Number of cells	Number of malignant cells	Percentage of cycling cells in the report	Percentage of cycling cells inferred using Cyclum
#79	896	468	1%	3%
#81	205	133	(low cycling)	11%
#80	480	125	(high cycling)	60%
#78	131	120	29%	42%
#88	351	117	(high cycling)	7%

For mel80, 81, and 88, percentages are not reported in the original report[7], but Figure S4C in the publication suggests whether or not they are high cycling.

Supplementary Note 1: mESC Dataset

Choosing dimensionality

Dimensionality of the embedding layer affects the ability of Cyclum to precisely capture the circular trajectory. Here, 11 dimensionalities (i.e., $k = 1, \dots, 11$) were tested. The bar plot in Supplementary Figure 2a shows that the tuning approach (detailed in Methods) chose $k^* = 2$ on the mESC dataset. The choice indeed resulted in better embedding (Supplementary Figure 2b) than that from a different choice $k^* = 1$ (Supplementary Figure 2c), further validating the tuning approach.

The tuning approach is readily available in Cyclum package. In practice, we suggest limiting k to five, (i.e., $k = 1, \dots, 5$), and utilizing the early stop option so that Cyclum will stop testing when the best choice has likely been found (e.g., for mESC, it will stop at $k = 3$, regardless of what the maximum k is allowed). Cyclum also allows a user to use a random subset of the data to determine the dimensionality before running the model on the whole dataset to accelerate the process.

By using the number where a circular component improves fitness of the model the most, Cyclum tracked the most prominent circular trajectory. Meanwhile, it is possible to observe multiple local maximum in the bar plot (e.g., $k = 4$ in Supplementary Figure 2a), which may indicate a less prominent circular process. User may also investigate those embeddings for a more comprehensive interpretation of the data.

Utilizing prior knowledge

As cell cycle is a relatively well annotated process, we attempted to use known cell-cycle genes acquired from gene ontology[8] to improve the results. Given the sparsity of single-cell sequencing data, and our observation that the cell cycle, albeit driven by a moderate set of genes, has a universal effect on many genes, we still strive to use all the information we have to retrieve its circular pattern. We find doubling the weight of these genes in the MSE loss function in the otherwise identical Cyclum model achieved a likely optimal balance of prior knowledge and *de novo* discovery in this particular experiment. Practically, this is done by doubling the expression value of the genes after the scaling. We observed that this approach increased the accuracy to 0.840 from 0.806 (a 4% improvement, Supplementary Figure 2d, f). It is worth noting that more weights did not lead to better performance. By only using the marker genes to infer the pseudotime (i.e., allocating infinite weights on them), the accuracy dropped to 0.799 (Supplementary Figure 2e). For technologies that produce noisier data than Smart-Seq, the adverse effect can be more significant. The extent and credibility of prior knowledge in other biological processes may also be significantly less than that of cell cycle. We suggest using the *de novo* approach in these cases.

Supplementary Note 2: Nicotine treated hESC Dataset

A recently published study on influence of nicotine on human embryonic stem cells (hESCs) used 10X droplet technology to sequence a nicotine treated sample and a control sample[9]. Sequencing and aligning retains 5,646 and 6,847 cells, respectively. We followed the filtering method used in the original article and kept 5,514 and 6,766 cells (i.e., 12,280 cells in total), but only performed a regular read-count normalization and gene scaling without regressing out any factors.

Cell-cycle pseudotime of cells

We first used Cyclum to predict pseudotimes for all the cells. Because the cells are not sorted by cell-cycle phases, we used GSEA to test if the pattern of cell cycle was well captured (see Methods: Evaluating accuracy of timings and separability of subclones). Results show that Cyclum outperforms reCAT, Cyclops, and the principal components (Supplementary Figure 8a). Oscope does not scale up and were not able to return a result within 120 hours, while reCAT took 44 hours to finish. In contrary, Cyclum were able to return results in 40 minutes (i.e., 66 times as fast) on a personal laptop computer equipped with a moderate NVidia GTX960M GPU. Only 1/6 of the time was used on the final training of the model, while most of the time was used to tune the model, which may also be done on a subset of the data to save more time. Furthermore, larger datasets will not further increase training time significantly, as neural network benefits from stochastic gradient decent and early stopping[10].

Circular magnitude of genes

We then ran Cyclum separately on the nicotine treated and the control sample. We retrieved the circular magnitude A_g of genes (detailed in Methods: Predicting marker) in each sample to compare (Supplementary Figure 8b). Most genes lie on the diagonal, showing that the circular magnitudes of most genes are comparable across datasets. A_g of gene *LDHA* in the treated group increased the most. It is a gene that regulates cell proliferation which is known to be downregulated post nicotine treatment[9]. It shows that A_g is a measurement independent to the expression level and benefits biological discovery. The second gene that shows significantly stronger circular pattern in the treated dataset is *ENO1*, a known target of nicotine, which plays roles in cell proliferation and non-small cell lung cancer, of which tobacco usage is a leading risk[11]. In addition, the A_g of a set of mitochondrial genes is decreased in the treated group, which also conforms to a recent report conjecturing mitochondria as a target of nicotine[12].

Supplementary Note 3: MET and EMT

EMT and its inverse process, the mesenchymal-epithelial transition (MET) are both essential to organogenesis[13]. Because different genes drive the two processes, the trajectory may be represented by a circle with two transition points corresponds to EMT and MET, respectively. To show that Cyclum can be used to study non-linear cycling processes other than cell-cycle, we performed preliminary analysis on an epithelial-mesenchymal transition (EMT) dataset[14] comprising 1,916 mESCs sequenced by Smart-seq2 protocol, among which 289 are epithelial cells and 352 are mesenchymal cells. Cyclum were able to construct a circular trajectory[14] (Supplementary Figure 9). Further analysis show that unique MET marker genes (i.e., genes that only incur MET but not EMT), *Cited1* and *Fzd7*[8], are identified in differential expression analysis when comparing the cells close to the two transition points. The circular embedding may further help identify the genes driving each process.

Supplementary Note 4: Math Background

Rationale of sinusoidal functions

Considering the feedbacks and interplays of genes, their expressions may be modeled using linear differential equations[15]. For a single factor (pseudo-time, e.g., cell cycle, senescence), the equation can be written as

$$\frac{d\mathbf{f}(t)}{dt} = \mathbf{A}\mathbf{f}(t),$$

where $\mathbf{f}(t)$ is a vector that varies over time. Each entry is the expressions of a gene, formally

$$\mathbf{f}(t) = \begin{bmatrix} f_1(t) \\ f_2(t) \\ \vdots \\ f_g(t) \end{bmatrix}.$$

This means the increasing/decreasing rate of gene expressions are decided by the combination of current gene expressions. The solution is generally a combination of sinusoidal and exponential functions. If we assume the only factor affecting the gene expressions is cell cycle, we expect the solution to be a sinusoidal function. For example, formula

$$\begin{bmatrix} f_1'(t) \\ f_2'(t) \end{bmatrix} = \begin{bmatrix} 0 & 1 \\ -1 & 0 \end{bmatrix} \begin{bmatrix} f_1(t) \\ f_2(t) \end{bmatrix}$$

means f_1 promotes transcription of f_2, while f_2 inhibits transcription of f_1. Notably, $\begin{bmatrix} f_1(t) \\ f_2(t) \end{bmatrix} = \begin{bmatrix} \sin t \\ \cos t \end{bmatrix}$ is one of the solutions, which means this kind of regulation will result in a circular process. This explains why sinusoidal functions model the genes well.

The time in the real world is always unidimensional, but cells on an inferred trajectory are not snapshots of one and the same cell. Thus, having more time axes (e.g., cell cycle and differentiation) allows for better modeling. In general cases, several processes may affect the gene expression, introducing multiple "timers" dictating the cell fate jointly. Nevertheless, we may mimic the separation of variables method for partial differential equations and assume that the (log-transformed) gene expressions can be separated into addable terms and still use ordinary differential equation components to find the solution for each term. The combination of linear and non-linear kernels is a realization of this idea (i.e. the summation of first and second order differential equations).

For example, we may have a partial differential equation with two time factors, s and t, the expression can be denoted as $\mathbf{f}(s,t)$, which subjects to

$$\frac{\partial \mathbf{f}(s,t)}{\partial s} + \frac{\partial \mathbf{f}(s,t)}{\partial t} = \mathbf{A}\mathbf{f}(s,t).$$

We further assume that $\mathbf{f}(s,t) = \mathbf{S}(s) + \mathbf{T}(t)$, where $\mathbf{S}(s)$ and $\mathbf{T}(t)$ only depend on s and t, respectively. This uses the "separation of variables." Then, we have

$$\frac{\partial \mathbf{S}(s)}{\partial s} + \frac{\partial \mathbf{T}(t)}{\partial t} = \mathbf{A}\big(\mathbf{S}(s) + \mathbf{T}(t)\big),$$

which can be solved for s and t individually. In other words, it can be written into two ordinary differential equations. This is roughly the background of having more than one dimension of pseudo-time in the Cyclum embedding.

Deconvolution

Ordinary differential equations can be depicted as a linear system. Cyclum infers pseudo-time over which the expression can be explained by Fourier expansion truncated to the first term. From the perspective of frequency domain, Cyclum eliminates that term to remove the cell-cycle factor. According to time-frequency duality, from the perspective of time domain, this is a deconvolution. The cell-cycle factors are removed by the deconvolution.

Supplementary Note 5: Simulation Studies

Choice of activation function in the encoder

The encoder part is less important than the decoder part where the sine and cosine functions, which guarantee the circular embedding, are located. Consequently, the choice of different activation functions is not expected to affect the ability of Cyclum in capturing the circular component. Nevertheless, we randomly simulated datasets that show circular trajectories to test hyperbolic tangent (tanh), rectified linear unit (ReLU), and scaled exponential linear unit (SeLU). We fixed the learning rate and used Glorot Normal (Xavier), He Normal, and LeCun Normal initializers, respectively, as each activation function relies on unique initializers to acheive its best performance. We noticed that tanh performs marginally better in more cases in terms of MSE (e.g., Supplementary Figure 10a, b), while the convergences rates of approaches are largely similar. Since the sizes of these neural networks are relatively small, the differences in time consumption across activation functions are inconsequential.

Influence of other nonlinear manifold in data

Single cell data may contain other types of nonlinear manifolds than circular. We expect them to be either captured by the linear component, if suggested by the tuning approach (see Methods), or left in the residual after removing the circular component. It is based on the fact that a nonlinear manifold can be precisely retained by linear components, with the tradeoff of using more dimensions than what is necessary for a proper nonlinear embedding. For example, it is common to perform PCA before t-SNE or UMAP embedding. The first 10 to 15 PCs tend to retain most of the information in the data, while t-SNE and UMAP further project the information in a two-dimensional space. In other words, PCA can, but must use more dimensions, to represent what could have been explained by only two dimensions. Cyclum uses only one dimension to explain the circular manifold, so that any other patterns, linear or nonlinear, will be retained in the residual, after removing the circular pattern.

To test the influence of other confounding nonlinear manifolds, we simulated a dataset containing both a circular and an additional nonlinear component using both sinusoid and sigmoid functions. Gene expression levels in single cells are determined by a sinusoidal circular pattern, a sigmoidal jump pattern, or both (via summation). We expect that with the help of one linear component, as suggested by our tuning approach, Cyclum can still accurately recover the time. If the model performed an accurate inference, we would observe that the predicted pseudotime match the simulated time well (manifest as a diagonal line) without being majorly affected by the jump pattern (the variance of which should be mainly captured by the additional linear component). The results we obtained largely matched our expectation, showing a diagonal curve in the circular pseudotime plot, with little displacement (Supplementary Figure 10c). The fidelity is comparable with the result on a solely sinusoidal dataset (Supplementary Figure 10d). Note that circular translation over X-axis is expected, as the start point of a circular trajectory is arbitrary. The sigmoid pattern is partially captured by the linear component, and partially left in the residue, without much effect on the circular embedding. Compared with the simulated datasets without the sigmoidal pattern, the tuning methods suggested to use one more linear component, validating the tuning approach.

Supplementary References

1. Chen, B., Ma, J., Li, C. & Wang, Y. Long noncoding RNA KCNQ1OT1 promotes proliferation and epithelial-mesenchymal transition by regulation of SMAD4 expression in lens epithelial cells. *Mol Med Rep* **18**, 16–24 (2018).
2. Guo, B., Zhang, Q., Wang, H., Chang, P. & Tao, K. KCNQ1OT1 promotes melanoma growth and metastasis. *Aging (Albany NY)* **10**, 632–644 (2018).
3. Sun, X. *et al.* Overexpression of long non-coding RNA KCNQ1OT1 is related to good prognosis via inhibiting cell proliferation in non-small cell lung cancer. *Thorac Cancer* **9**, 523–531 (2018).
4. Zhang, S. *et al.* LncRNA KCNQ1OT1 regulates proliferation and cisplatin resistance in tongue cancer via miR-211-5p mediated Ezrin/Fak/Src signaling. *Cell Death & Disease* **9**, 742 (2018).
5. Bai, N. *et al.* circFBLIM1 act as a ceRNA to promote hepatocellular cancer progression by sponging miR-346. *Journal of Experimental & Clinical Cancer Research* **37**, 172 (2018).
6. Toeda, Y. *et al.* FBLIM1 enhances oral cancer malignancy via modulation of the epidermal growth factor receptor pathway. *Mol. Carcinog.* **57**, 1690–1697 (2018).
7. Tirosh, I. *et al.* Dissecting the multicellular ecosystem of metastatic melanoma by single-cell RNA-seq. *Science* **352**, 189–196 (2016).
8. Bult, C. J. *et al.* Mouse Genome Database (MGD) 2019. *Nucleic Acids Res* **47**, D801–D806 (2019).
9. Guo, H. *et al.* Single-cell RNA sequencing of human embryonic stem cell differentiation delineates adverse effects of nicotine on embryonic development. *Stem Cell Reports* **12**, 772–786 (2019).
10. Lopez, R., Regier, J., Cole, M. B., Jordan, M. I. & Yosef, N. Deep generative modeling for single-cell transcriptomics. *Nat Methods* **15**, 1053–1058 (2018).
11. Li, M. D. *Tobacco Smoking Addiction: Epidemiology, Genetics, Mechanisms, and Treatment*. (Springer Singapore, 2018).
12. Malińska, D. *et al.* Mitochondria as a possible target for nicotine action. *J Bioenerg Biomembr* **51**, 259–276 (2019).
13. Thiery, J. P., Acloque, H., Huang, R. Y. J. & Nieto, M. A. Epithelial-mesenchymal transitions in development and disease. *Cell* **139**, 871–890 (2009).
14. Dong, J. *et al.* Single-cell RNA-seq analysis unveils a prevalent epithelial/mesenchymal hybrid state during mouse organogenesis. *Genome Biology* **19**, 31 (2018).
15. Chen, T., He, H. L. & Church, G. M. Modeling gene expression with differential equations. *Pac Symp Biocomput* 29–40 (1999).
