# Supplementary material for: Latent periodic process inference from single-cell RNA-seq data
Source: Nat Commun. 2020 Mar 18;11:1441. doi: 10.1038/s41467-020-15295-9 (PMC7080821; doi:10.1038/s41467-020-15295-9)
Supplement: Supplementary file 3 — Description of Additional Supplementary Files [file 41467_2020_15295_MOESM3_ESM.pdf]

Description of Additional Supplementary Files

File Name: Supplementary Data 1
Description: Cell-cycle marker genes related to simulating virtual tumors.
